# Crumbling Pathogenesis and Biomarkers for Diabetic Peripheral Neuropathy

**DOI:** 10.3390/biomedicines13020413

**Published:** 2025-02-08

**Authors:** Zhao Zhong Chong, Nizar Souayah

**Affiliations:** 1 Department of Neurology, New Jersey Medical School, Rutgers University, 185 S. Orange Ave, Newark, NJ 07103, USA; 2 Department of Neurology, New Jersey Medical School, Rutgers University, 90 Bergen Street DOC 8100, Newark, NJ 07101, USA

**Keywords:** diabetic neuropathy, oxidative stress, inflammation, biomarker, nerve conduction

## Abstract

**Background**: Diabetic sensorimotor polyneuropathy (DSP) is a common chronic diabetic complication. Traditionally, DSP was once considered irreversible with a typical loss of axon. However, the superimpose of acquired demyelination on axonal loss in DSP patients has been observed, implying that DSP may be preventable or reversible, particularly within a subgroup of patients exhibiting early-stage acquired demyelination, underscoring the critical importance of identifying early prognostic markers. **Methods**: We systemically review the literature on the roles of biomarkers in predicting DSP and monitoring the progress. The underlying mechanisms of biomarkers were also discussed. **Results**: The pathogenesis of DSP is multifaceted, with various pathological mechanisms contributing to its development. Key mechanisms include aberrant glucose metabolism and induction of oxidative stress and inflammation. Several pathological processes, such as disrupted glucose metabolism, nerve damage, impaired microcirculation, genetic variants, and microRNA dysregulation, lead to molecular and protein changes that may be detectable in blood and other biological compartments, thus serving as potential biomarkers for DSP progression. However, the utility of a biomarker depends on its predictive accuracy, practicality, and ease of measurement. **Conclusions**: Most biomarkers for predicting DSP have demonstrated suboptimal predictive value, and many lack established accuracy in forecasting DSP progression. Consequently, the diagnostic utility of any single biomarker remains limited. A comprehensive combination of biomarkers from various categories may hold incredible promise for accurate detection. As artificial intelligence (AI) techniques, especially machine learning, rapidly advance, these technologies may offer significant potential for developing diagnostic platforms to integrate and interpret complex biomarker data for DSP.

## 1. Introduction

### 1.1. The Critical Early Diagnosis for Diabetic Neuropathy

Diabetic sensorimotor polyneuropathy (DSP) is one of the common chronic complications of diabetes mellitus (DM). DSP predisposes to the formation of foot ulcers and causes limb amputation [1,2]. The nerve damage in DSP commonly manifests as distal, bilateral, and symmetric damage to the nerves of the lower limbs, often referred to as stocking-glove neuropathy. In the early stages, sensory symptoms such as numbness, pain, tingling, and paresthesias are predominant, while motor symptoms, including limb weakness, typically emerge later.

Peripheral nerves can be either myelinated or unmyelinated. Myelinated nerves have faster conduction speed and carry motor, touch, and proprioceptive impulses, while unmyelinated nerves have a slower conduction velocity and carry pain, temperature, and autonomic impulses. However, most nerves are mixtures of myelinated and nonmyelinated fibers. Peripheral nerve demyelination caused by the loss of myelin typically leads to nerve conduction slowing (NCS) and functional deficit.

The axonal loss has been considered the primary nerve damage in DSP; however, NCS that is independent of compound muscle action potential (CMAP) has been identified [3]. The sole demyelination of the proximal nerve root has been observed in autopsies of DSP patients [4]. The NCS resulting from the sole demyelination was CMAP amplitude-independent motor NCS in diabetic DSP [3]. Clinical examination and conventional nerve conduction study criteria cannot clearly identify minor degrees of demyelination with pure loss of large fibers until it reaches a severity well beyond the usual spectrum of pure axonal loss. However, demyelination in peripheral nerves is potentially reversible, and early identification of demyelination could benefit patients by enabling timely interventions to improve their quality of life. Therefore, developing new diagnostic tools that detect early, mild demyelination as an initial sign of neuropathy should be a priority.

We previously established regression analysis to develop confidence intervals that assess the range of conduction slowing from primary demyelination in patients with chronic inflammatory demyelinating polyneuropathy (CIDP) [5]. The regression equations were later used to analyze NCS in patients with DSP. The results indicated that the regression analysis could identify the NCS in DSP beyond axonal loss [6]. However, the requirement for electrodiagnostic equipment and complicated analysis is not feasible for most clinics.

### 1.2. Identifying Critical Risk Factors

The most critical risk factor for DSP has been explored. Previously, glycemic control was considered closely linked to the severity of DSP based on morphological criteria in both type 1 and type 2 diabetes mellitus (T1DM and T2DM) patients [7]. The glycosylated hemoglobin (HbA1c) level was used as a glycemic control criterion. The density of nerve fiber and nerve conduction velocity (NCV) significantly differed in patients with HbA1c over and below 9 [7]. Tight glycemic control had once been proposed as the critical measurement to stop the development of neuropathy in diabetic patients [8]. Glycemic control by multiple insulin injections significantly delays the onset and progression of DSP in Japanese patients with T2DM [9,10]. Most continuous glucose monitoring-derived glycemic variability metrics were significantly associated with sural NCS [11,12]. However, some studies failed to reproduce the findings [13,14].

Although HbA1c is routinely used to evaluate a person’s level of glucose control, it only reflects an average of the blood sugar level over the past three months. The assessment of glycemic control by HbA1c fails to capture the interday or intraday glucose variations [11]. In this regard, increased HbA1c variability was later assessed in the prognosis of DSP in T2DM patients. HbA1c variability is a better parameter for assessing glucose variability. The increased HbA1c variability has been found in T2DM patients with DSP. The sensitivity and specificity of HbA1c variability for predicting DSP were 66.67% and 65.73%, respectively, suggesting that HbA1c variability could predict DSP in T2DM patients [15].

Lately, glycemic variability has been identified as a crucial determinant for the progressive development of DSP. In addition, glycemic variability was suggested as a risk factor independent of other factors for DSP in T2DM patients since HbA1c was not correlated with glycemic variability in patients with HbA1c levels below 7.0% in T2DM patients [16,17]. More significant glycemic variability was observed in T2DM patients with DSP. Moreover, multivariate logistic regression analysis demonstrated that the mean of daily differences is the most significantly independent risk factor for DSP [18]. Two meta-analysis studies, including nine studies (a total of 3649 patients) and ten studies (a total of 72,565 patients), respectively, strongly support that higher glucose variability promotes the development of DSP in patients with either T1DM or T2DM [19,20].

### 1.3. Beneficial Promise of Biomarkers for DSP

DSP is a complex condition that involves multiple pathological processes, including aberrant glucose metabolism, nerve damage, microcirculation impairment, genetic predisposition, and dysregulation of microRNA expression. These processes induce alterations in proteins and molecules, which may be detectable in blood, cerebrospinal fluid, or other bodily compartments. Such molecules may serve as biomarkers for tracking the progression of DSP. However, despite advances in understanding DSP, there remains a critical lack of reliable diagnostic parameters capable of identifying the early stages of DSP. The discovery of biomarkers that can effectively monitor disease progression and predict outcomes in early, potentially reversible stages of DSP is pivotal. These biomarkers could enable timely interventions to prevent or mitigate the irreversible damage associated with axonal loss. For example, implementing early symptomatic treatments has demonstrated tangible benefits in managing DSP. A notable multicenter study illustrated the therapeutic potential of intravenous immunoglobulin (IVIG) in DSP patients, showing improvements in CMAP amplitude and NCV. Furthermore, a prospective study demonstrated that IVIG significantly alleviated pain in patients with DSP [21]. The findings underscore that the condition is at least symptomatically reversible when detected and treated before axonal degeneration becomes irreversible.

Identifying biomarkers for the early phase of DSP or mild demyelination is thus particularly critical. Such biomarkers could offer valuable diagnostic and prognostic insights, aiding clinicians in implementing timely therapeutic interventions. Emerging studies aim to identify and validate a wide array of biomarkers based on the underlying pathogenesis of DSP. These include markers of oxidative stress, inflammation, nerve injury, and endothelial dysfunction, among others.

This review consolidates the progress in biomarker research, summarizing their roles in early detection, disease monitoring, and therapeutic response in DSP. This review covers the recent progress in developing biomarkers for diabetic neuropathy. The article summarized different markers, illustrating their contribution to the development of DSP, to gain insight into the mechanisms underlying the roles of these biomarkers. As the field advances, identifying specific and sensitive biomarkers could revolutionize the clinical management of DSP, enabling early diagnosis and improved prognostic accuracy, ultimately improving patient outcomes.

## 2. Oxidative Stress Associated Pathogenesis and Biomarkers

### 2.1. Oxidative Stress

Oxidative stress results from the excessive generation of free radicals that surpass the neutralizing ability of antioxidants in the body. The common free radicals are reactive oxygen species (ROSs), including superoxide anion, hydrogen peroxide, peroxyl radical, hydroxyl radical, hydrogen peroxide, and oxygen anion. Free radicals also include reactive nitrogen species, such as nitric oxide (NO) and peroxynitrite anion.

The endogenous antioxidants that function to eradicate the free radicals include scavenging enzymes (superoxide dismutase, catalase, glutathione peroxidase, and glutathione S-transferase) and non-enzymatic substances (albumin, bilirubin, glutathione, alpha-lipoic acid, coenzyme Q, ferritin, metallothioneine, and L-carnitine). Under physiological conditions, small amounts of ROSs are scavenged by endogenous antioxidant systems.

Overproduced ROSs interact with lipids, amino acids, DNA, and glucose, leading to the peroxidation of lipids, damage of protein and DNA, and glycation. Oxidative stress has been implicated in various diseases, including diabetes and associated complications, obesity, cardiovascular diseases, and neurodegenerative diseases [22].

### 2.2. Induction of Oxidative Stress Under Hyperglycemia

Hyperglycemia can trigger aberrant metabolic pathways that increase the production of ROSs and promote oxidative stress. Under hyperglycemic conditions, excessive glucose enters alternative metabolism pathways. More glucose enters glycolysis, increasing NADH by reducing NAD^+^. In the mitochondria, the excessive NADH is oxidized by mitochondrial electron transport chain complex 1, generating excessive superoxide ions. Increasing superoxide ions induces oxidative stress and subsequently decreases the activity of glyceraldehyde-3-phosphate dehydrogenase (GAPDH), resulting in the inhibition of glycolysis. This will propel the glucose products into alternative metabolism pathways, including the polyol and hexose monophosphate (HMP) pathways, resulting in the activation of protein kinase C (PKC) and increased production of ROSs [23,24].

**Figure 1:**
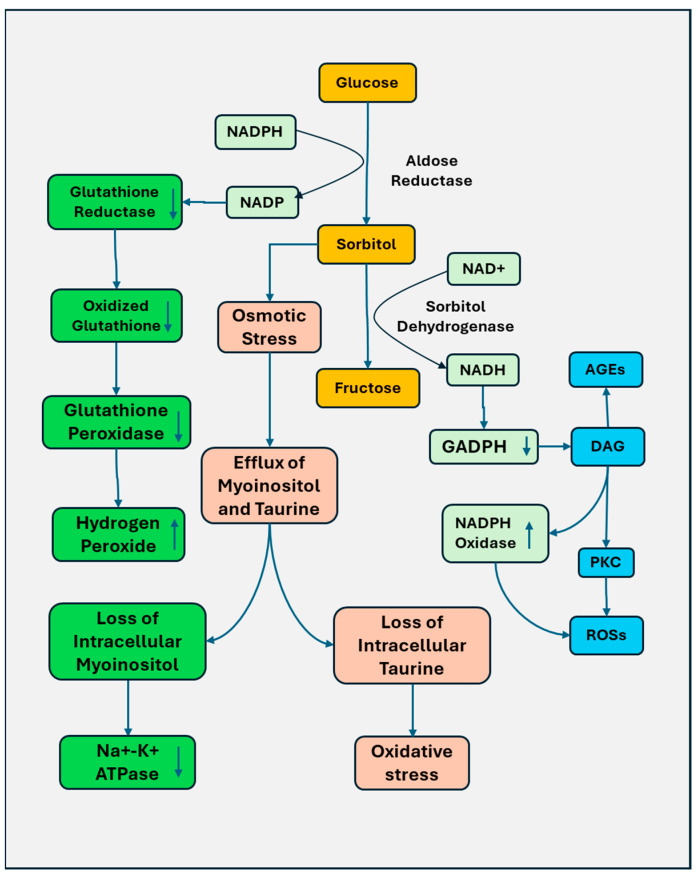
The polyol pathway and oxidative stress induced by high glucose. Hyperglycemia promotes the binding affinity to aldose reductase, converting glucose to sorbitol, which is then converted to fructose by sorbitol dehydrogenase. The processes consume nicotinamide adenine dinucleotide phosphate (NADPH). The depletion of NADPH leads to a decreased activity of glutathione reductase, increasing the accumulation of oxidized glutathione and subsequently decreasing the activity of glutathione peroxidase, leading to its inability to reduce hydrogen peroxide. Increased sorbitol causes osmotic stress and compensatory efflux of myoinositol and taurine. Loss of cellular myoinositol impairs the activity of Na^+^-K^+^ ATPase. The loss of taurine leads to increased oxidative stress. In addition, during the reaction of sorbitol dehydrogenase, NAD^+^ is oxidized to NADH, and the increased ratio of NADH/NAD^+^ inhibits GAPDH and subsequently leads to the accumulation of glycolytic precursor diacylglycerol (DAG). DAG triggers the production of advanced glycation end products (AGEs) and ROS by activating NADPH oxidase and PKC.

The polyol pathway is induced by excessive glucose. Hyperglycemia increases the binding affinity to aldose reductase, converting glucose to sorbitol, which is then converted to fructose by sorbitol dehydrogenase (Figure 1). The processes consume nicotinamide adenine dinucleotide phosphate (NADPH), the depletion of which reduces the activity of glutathione reductase, resulting in the accumulation of oxidized glutathione and a subsequent decrease in the activity of glutathione peroxidase (GPX). The oxidative stress consequently results since reduced glutathione is essential for GPX to reduce hydrogen peroxide. Increased sorbitol causes osmotic stress, resulting in compensatory efflux of myoinositol and taurine. Loss of cellular myoinositol impairs the activity of sodium/potassium Na^+^-K^+^ ATPase. The loss of taurine leads to increased oxidative stress. In addition, during the reaction of sorbitol dehydrogenase, NAD^+^ is oxidized to NADH, and the increased ratio of NADH/NAD^+^ inhibits GAPDH and subsequent accumulation of glycolytic precursor diacylglycerol (DAG). DAG triggers the production of advanced glycation end products (AGEs) and ROS by activating NADPH oxidase and PKC [25]. The accumulation of fructose also induces glycation and the formation of AGEs, contributing to increased oxidative stress [26].

The activation of the polyol pathway was observed in diabetic rats. Proteomics and metabolomics analysis showed that glucose and polyol pathway intermediates were significantly enhanced in the peripheral nerves and dorsal root ganglia (DRG) in streptozotocin (STZ)-diabetic rats. However, a significant increase in mitochondrial oxidative phosphorylation and perturbation of lipid metabolism was only found in the sciatic nerve, suggesting that peripheral nerves are more likely to be subjected to metabolic disturbance of diabetes [27].

In contrast, inhibition of the polyol pathway shows improved outcomes. The aldose reductase inhibition attenuated diabetes-caused behavioral deficits, improved nerve conduction, and reduced nerve structure damage [28]. Genetic manipulation of aldose reductase has also been studied in animal models. T1DM mice with overexpression of aldose reductase had a significant decrease in tibial NCV compared to non-transgenic mice [29]. In aldose reductase knockout mice, no depletion of reduced glutathione, increased superoxide formation, and DNA damage in the sciatic nerve were observed [30].

Most early trials that evaluate the efficacy of aldose reductase inhibitors for diabetic polyneuropathy did not show beneficial effects on nerve conduction parameters [31]. However, aldose reductase inhibitors are still promising for treating DSP, and the more carefully designed trials for well-selected aldose reductase inhibitors may improve the outcomes. Epalrestat, an aldose reductase inhibitor, has been shown to reduce sorbitol levels in the sciatic nerve [32]. In T2DM patients, the application of epalrestat significantly prevented the NCS in the tibial nerve and sural nerve [33].

The HMP shunt, also known as the pentose phosphate pathway, is an alternative pathway to glycolysis. The glycolysis intermediates, including glucose 6-phosphate, fructose 6-phosphate, and glyceraldehyde 3-phosphate, are used in this pathway to produce ribose-5-phosphate and NADPH. Glutamine fructose-6-phosphatase converts fructose-6-phosphate into glucosamine-6 phosphate and subsequently into uridine 5-diphosphate-N-acetylglucosamine (GlcNac). GlcNac can induce the activation of specificity protein 1 (Sp1), which upregulates the gene transcription of both transforming growth factor-β1 (TGF-β1) and plasminogen activator inhibitor-1 (PAI-1) [34]. Dihydroxy-acetone phosphate is another glycolytic intermediate converted to DAG in the HMP pathway. DAG subsequently activates PKC.

**Figure 2:**
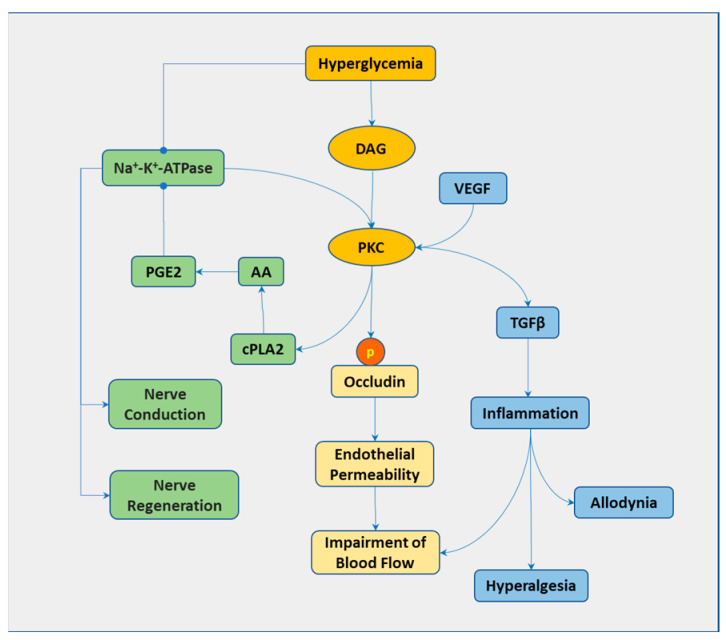
The roles of protein kinase C (PKC) in DPN. Hyperglycemia-induced alternative glucose metabolic pathways lead to diacylglycerol (DAG) accumulation, which then activates PKC. Hyperglycemia-induced inhibition of Na^+^-K^+^-ATPase also results PKC activation. Active PKC stimulates cytosolic phospholipase A2 (cPLA2), which breaks down the phospholipids and liberates arachidonic acid (AA), leading to an increased production of PGE2. PGE2 inhibits the activity of Na^+^-K^+^-ATPase. The inhibition of Na^+^-K^+^-ATPase has been associated with NCS and impaired nerve regeneration. In addition, PKC can induce the phosphorylation of the tight junction protein occludin, promoting vascular endothelial growth factor (VEGF)-induced increase in endothelial permeability, leading to impaired blood flow to the nerves. PKC-mediated transforming growth factor-β (TGF-β) regulates diabetic neuropathy with neuroinflammation and is associated with hyperalgesia and allodynia.

PKC is involved in a variety of signaling pathways (Figure 2). Hyperglycemia-induced inhibition of Na^+^-K^+^-ATPase is associated with PKC activation. Elevated glucose sequentially activates PKC and cytosolic phospholipase A2 (cPLA2), which breaks down the phospholipids and liberates arachidonic acid. This results in an increased production of PGE2, which inhibits the activity of Na^+^-K^+^-ATPase [35]. The inhibition of Na^+^-K^+^-ATPase has been associated with NCS and impaired nerve regeneration [36]. PKC-induced phosphorylation of occludin, a tight-junction protein, is essential for vascular endothelial growth factor (VEGF)-induced endothelial permeability [37], leading to impaired blood flow to the nerves. PKC-mediated TGF-β regulates diabetic neuropathy with neuroinflammation and is associated with hyperalgesia and allodynia [38]. Inhibition of PKCβ can correct both the sciatic motor and saphenous nerve sensory NCV and blood flow to the sciatic nerve and superior cervical ganglion in T1DM rats [39]. PKC is also involved in the high glucose-induced generation of ROS by activating NADPH oxidase [40].

### 2.3. Oxidative Stress Biomarkers

Potential oxidative stress biomarkers for DSP have been explored. The link between these markers and the risk for DSP has been investigated in diabetic patients.

#### 2.3.1. Thiol-Disulfide Balance

Visfatin (nicotinamide phosphoribosyltransferase) is an adipokine produced by fat cells and functions in cell proliferation and NAD^+^ synthesis. In T2DM patients, the levels of serum glucose, HbA1c, and visfatin all have a positive correlation with the total oxidative stress index [41]. In contrast, total and native plasma thiol antioxidants, including albumin, cysteine, cysteinylglycine, glutathione, homocysteine, and γ-glutamylcysteine, were negatively correlated with oxidant levels [41]. Both native and total levels of thiol of the DSP groups were significantly decreased compared to the control group. Oxidative stress induced the transformation of thiols into disulfide forms. The disulfide in DSP patients was increased considerably, and the impairment of thiol-disulfide balance was observed [42,43].

In addition, a significant decrease of superoxide dismutase (SOD) was observed in T2DM patients with neuropathy. The level of oxidative DNA damage marker was significantly increased in T2DM patients with DSP compared to both T2DM patients without DSP and controls [44].

#### 2.3.2. Malondialdehyde

Malondialdehyde (MDA) is a reactive product from lipid peroxidation by ROSs and is one of the most used biomarkers for oxidative stress. High serum MDA levels have been demonstrated in patients with DSP [45] and significantly correlated with the DSP score, calculated by the history score in Michigan Neuropathy Screening Instrument (MNSI), 7 or higher, and the physical assessment score in MNSI, more than 2 [46]. Applying *N*-acetylcysteine (NAC) significantly reduced painful scores in DSP patients. Simultaneously, a significant decrease in serum MDA and increased serum levels of antioxidants were observed [47]. Taurine, a potent antioxidant, significantly improves SOD activity and decreases MDA concentration in the spinal cord of diabetic rats [48].

**Figure 3:**
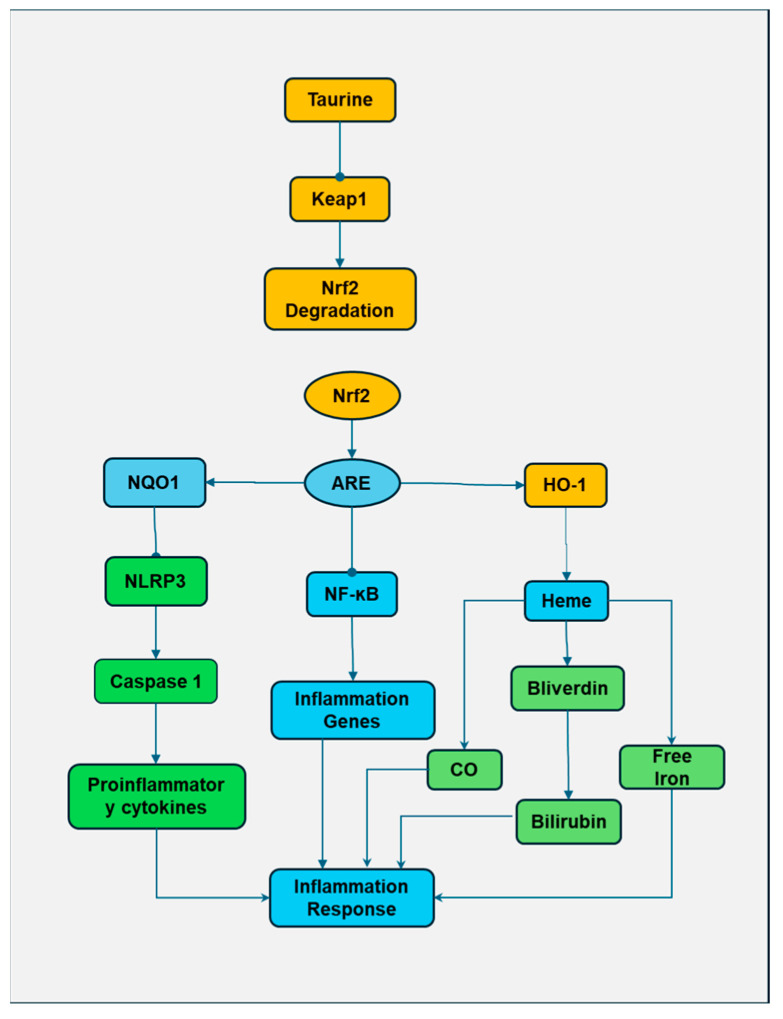
Antioxidant effect of taurine. The effects of taurine may be regulated through Kelch ECH-associating protein 1 (Keap1)-nuclear factor erythroid 2-related factor 2 (Nrf2) cell signaling pathway. Keap1 is a repressor protein that binds to Nrf2 and promotes its degradation through the ubiquitin-proteasome pathway. Taurine reduces the level of Keap1, leading to increased Nrf2 and heme oxygenase (HO-1) levels. Nrf2 binds to the antioxidant response element (ARE) of its target genes to mediate gene expression of pro-inflammatory cytokines. Nrf2 induces the expression of HO-1 and increases the activity of HO-1. HO-1 catalyzes the degradation of heme into carbon monoxide (CO), free iron, and biliverdin, which then is converted to bilirubin by biliverdin reductase. Free heme is pro-inflammatory, while CO, bilirubin, and HO-1 have significant anti-inflammatory effects. Moreover, Nrf2 has been shown to induce the quinone oxidoreductase (NQO1) expression, which inhibits the activation of NOD-like receptor protein 3 (NLRP3) inflammasome. NLRP3 regulates the maturation and secretion of pro-inflammatory cytokines via activating caspase 1. Nrf2 also inhibits NF-ĸB transcriptional activity.

The antioxidant effect of taurine may be regulated through Kelch ECH associating protein 1 (Keap1)-nuclear factor erythroid 2-related factor 2 (Nrf2) cell signaling pathway since taurine downregulated the level of Keap1 mRNA and upregulated Nrf2 and heme oxygenase-1 (HO-1) mRNA levels (Figure 3) [48]. The Keap1-Nrf2 signaling pathway regulates the defense against oxidative stress in cells. Nrf2 is a redox-sensitive transcription factor that binds to the antioxidant response element (ARE) of its target genes, while Keap1 binds to Nrf2, leading to Nrf2 degradation [49]. HO-1 is an enzyme that breaks down heme and has antioxidant and anti-inflammatory properties [50]. Nrf2 increases the HO-1 expression [51]. HO-1 converts the heme into carbon monoxide (CO), free iron, and biliverdin, which is further catalyzed to bilirubin by biliverdin reductase. Free heme has pro-inflammatory properties; CO, bilirubin, and HO-1 are anti-inflammatory molecules [52]. Moreover, Nrf2 induces the expression of the quinone oxidoreductase (NQO1), inhibiting the activation of NOD-like receptor protein 3 (NLRP3) inflammasome [53]. NLRP3 regulates the maturation and secretion of pro-inflammatory cytokines via activating caspase 1 to process the activation of pro-inflammatory cytokines [54]. In addition, Nrf2 can repress nuclear factor (NF)-ĸB transcriptional activity [55].

#### 2.3.3. Poly (ADP-ribose) Polymerase (PARP)

PARP is an enzyme in DNA repair, mitochondrial homeostasis, and apoptosis. Hyperglycemia induces the activation of PARP caused by ROS-induced DNA damage [56]. The hyperactivated PARP turns NAD^+^ into nicotinic acid and ADP-ribose, leading to the depletion of NAD^+^ and ATP. The accumulation of ADP-ribose polymer slows down glycolysis and impairs mitochondrial respiration. ADP-ribose polymer translocates to mitochondria, releasing apoptosis-inducing factor (AIF). AIF is translocated to the nucleus to induce PARP-dependent cell death [57].

In experimental animals, PARP activation promoted the formation of ROSs in the peripheral nerve of T1DM rats and human Schwann cells exposed to elevated glucose [58]. PARP inhibitor 1,5-isoquinolinediol reduced superoxide formation in sciatic nerve and attenuated hyperalgesia, decreased tactile allodynia, and improved behavior in STZ-induced diabetic rats [59]. Applying 3-aminobenzamide and 1,5-isoquinolinediol, both PARP inhibitors, improved nerve blood flow, increased NCV, and attenuated energy failure in STZ-induced diabetic rats [60]. PARP-1 increases the generation of mitochondrial ROS and induces cell death via PARylation of activating transcription factor-4, which upregulates the expression of MAP kinase phosphatase-1 and subsequently activates MAP kinases (p38/JNK) [61].

PARP also triggers the activation of NF-κB. PARP interacts directly with both subunits of NF-κB, p50 and p65, and high glucose increases the PARP/p50 complex in the retinal cells [62]. PARP also induces the activation of activator protein 1 (AP-1) and nuclear factor of activated T cells (NFAT), leading to the release of inflammatory cytokines and chemokines [63].

## 3. Inflammation Associated Pathogenesis and Biomarkers

### 3.1. The Induction of Inflammation

DSP and its associated pain have been strongly associated with inflammatory response [64]. As mentioned above, hyperglycemia induces oxidative stress. Oxidative stress initiates a series of cascade pathways to increase the gene transcription of proinflammatory cytokines [54,65].

**Figure 4:**
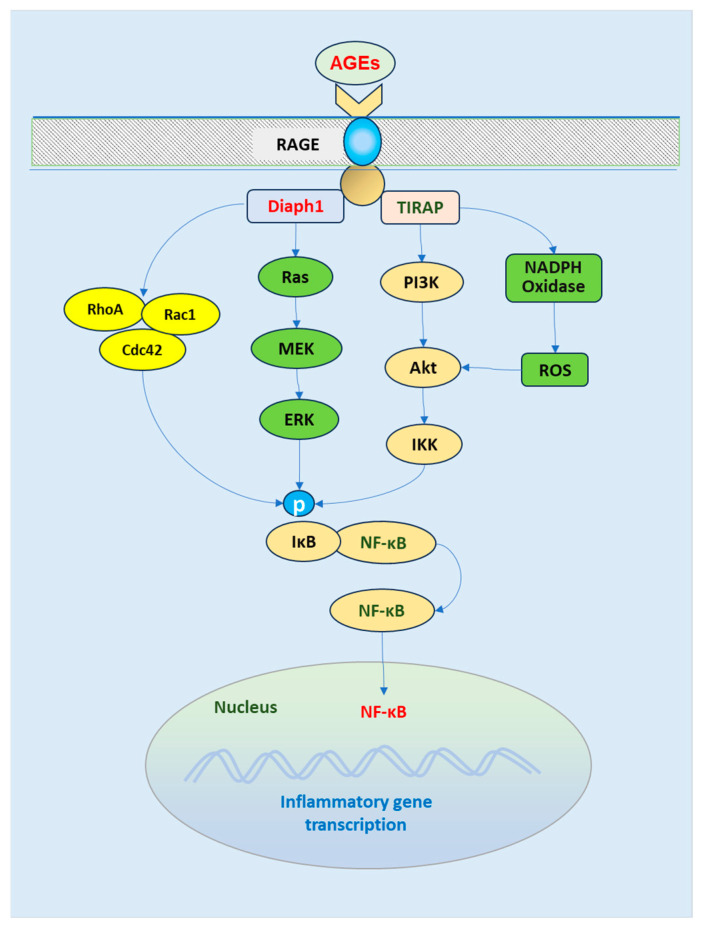
Advanced glycation end products (AGEs) induced inflammatory cell signaling pathways. Hyperglycemia increases the production of AGEs. AGEs interact with receptors for AGEs (RAGE), resulting in the binding of the RAGE intracellular domain to diaphanous-related formin1 (Diaph1) and toll-interleukin 1 receptor domain-containing adaptor protein (TIRAP). Several downstream signaling pathways can induce NF-κB activation, including the activation of phosphatidylinositol-3-kinase (PI3K)/Akt results in the phosphorylation of IκB kinase (IKK), which phosphorylates IκB, releasing NF-κB from its IκB binding and leading to subsequent translocation of NF-κB into the nucleus. NADPH oxidase/reactive oxidative species (ROS) also activate IKK. In addition, AGE/RAGE induces the activation of Ras-mitogen-activated protein kinase (MAPK) kinase (MEK)/extracellular signal-regulated kinase (ERK). ERK can phosphorylate IκB. Small Rho GTPase proteins, including homolog family member A (RhoA), cell division control protein 42 (Cdc42), and RAS-related C3 botulinum toxin substrate 1 (Rac1), promote the activation of NF-κB by phosphorylating IκB. NF-κB in the nucleus promotes the gene transcription of proinflammatory cytokines.

AGEs also induce cell signaling pathways to promote inflammatory response (Figure 4). Long-term hyperglycemia increases the production of AGEs. AGEs interact with receptors for AGEs (RAGE) to induce intracellular signaling pathways, releasing various inflammatory cytokines [66]. The AGE–RAGE interaction induces the RAGE intracellular domain to bind to diaphanous-related formin1 (Diaph1) and toll-interleukin 1 receptor domain-containing adaptor protein (TIRAP), leading to the activation of NF-κB. Several downstream signaling pathways can induce NF-κB activation [67,68,69]. The activation of phosphatidylinositol-3-kinase (PI3K)/Akt results in the phosphorylation of I-κB kinase (IKK), which phosphorylates I-κB, releasing NF-κB from its binding with I-κB. Free NF-κB translocation into the nucleus occurs. NADPH oxidase/ROS also activates IKK [70]. In addition, AGE/RAGE induces the activation of Ras-mitogen-activated protein kinase (MAPK) kinase (MEK)/extracellular signal-regulated kinase (ERK). ERK can phosphorylate I-κB and promote the nuclear translocation of NF-κB. Small Rho GTPase proteins, including homolog family member A, cell division control protein 42, and RAS-related C3 botulinum toxin substrate 1, also promote NF-B activation [71]. NF-κB in the nucleus induces the gene transcription of proinflammatory cytokines [72].

The response of inflammatory cells and proinflammatory cytokines have been elucidated in DSP, and many associated molecules have been considered biomarkers of DM and DSP.

### 3.2. Inflammatory Biomarkers

#### 3.2.1. Macrophages

Macrophage infiltration contributes to diabetic neuropathy. Monocytes can be differentiated upon stimulus into pro-inflammatory (M1) or anti-inflammatory (M2) macrophages. Pro-inflammatory M1 macrophage infiltration and increased pro-inflammatory cytokine expression have been demonstrated in diabetic animals, which is involved in diabetic nerve injury. In T1DM and T2DM mice, the intraneural migration of macrophages and T cells in the peripheral nerves was observed [73,74,75]. Concomitant endoneurial expression of IL-1β in the sciatic nerve of experimental DSP was significantly increased in Lewis rats with STZ injection [76]. The macrophage infiltration in the sciatic nerve of hyperglycemic ob/ob mice was associated with nerve fiber loss [77]. Hyperoside, an active component found in the Chinese herbal Tu-Si-Zi, has been found to mediate macrophage polarization from M1 to M2 both in vivo and in vitro, accompanied by reduced expression of monocyte chemoattractant protein-1 (MCP-1), tumor necrosis factor (TNF)-α, and inducible nitric oxide synthase (iNOS) [78], suggesting its potential in the management of DSP.

#### 3.2.2. Neutrophil-to-Lymphocyte Ratio

The neutrophil-to-lymphocyte ratio (NLR) is a chronic inflammatory biomarker. T2DM patients with higher NLR levels are more likely to develop DSP [79,80]. The NLR levels were significantly higher in the DSP patients with T2DM than in patients with T2DM only. Multivariate logistic regression analysis illustrated that NLR correlated with the development of DSP. The accuracy of NLR in predicting the onset of DSP reached 65.41%, suggesting that detecting NLR might benefit the early diagnosis of DSP [81]. In addition, platelet-to-lymphocyte ratio (PLR) has been found to have similar prognostic values of DSP in T1DM patients [80]. Interestingly, HbA1c and NLR have some synergic effects on predicting DSP. In one clinical study, the sensitivity of HbA1c, NLR, and their combination for predicting DSP is 71.6%, 90.0%, and 97.2%, respectively [82].

#### 3.2.3. NF-κB

The NF-κB family comprises p50/p105, p52/p100, RelA, RelB, and c-Rel. In its inactive condition, RelA, p50 c-Rel, and RelB bind to I-κB and are sequestered in the cytoplasm. Phosphorylation and degradation of I-κB release NF-κB from the binding and facilitate its translocation to the nucleus. NF-κB then regulates the transcription of pro-inflammatory genes.

Increased activation of NF-kB was shown in DM. In T1DM monocytes of patients, RelA and RelB were constitutively activated, and overexpression of the src homology 2 domain-containing protein tyrosine phosphatase-1 (SHP-1), which negatively regulates NF-kB, was identified, which may reflect a defense response [83]. In T2DM patients with DSP, higher serum NF-κB levels were observed than in T2DM patients without neuropathy. The serum NF-κB levels positively correlated with total neuropathy score in the DSP patients [84].

In contrast, antagonizing NF-κB attenuates the inflammatory response and improves diabetic neuropathy. In T2DM mice, oral administration of catalpol significantly improved fasting insulin levels and glucose tolerance. It decreases the expression of inflammatory genes in adipose via suppressing c-Jun NH2-terminal kinase (JNK) and NF-κB [85]. Curcumin derived from turmeric interacts with NF-κB, decreasing inflammatory cytokines and improving β-cells function in T1DM in rats [86]. Melatonin decreases NF-κB, leading to decreased TNF-α, IL-6, iNOS, and COX-2 in sciatic nerves and improved motor nerve conduction and nerve blood flow in the rat model of diabetes [87]. Another NF-κB inhibitor, JSH-23 (6-methyl-N1-(3-phenyl-propyl)-benzene-1,2-diamine), also lowered the levels of IL-6, TNF-α, COX-2, and iNOS and attenuated functional deficits in diabetic rats [88].

#### 3.2.4. Toll-like Receptors

Toll-like receptors (TLRs) are the major receptors for pathogen-associated molecular patterns (PAMPs) and endogenous damage-associated molecular patterns (DAMPs). Ten functional TLRs have been identified in humans (TLR1–10) [89]. TLRs are closely related to the induction of inflammatory cytokine release. TLRs are type I transmembrane proteins that contain three structural domains: an extracellular leucine-rich repeats (LRRs) motif, a transmembrane domain, and a cytoplasmic Toll/IL-1R (TIR) domain. The LRRs motif recognizes PAMPs, while the TIR domain associates with signal adaptors to initiate downstream signaling pathways [90].

Activated TLRs recruit TIR domain-containing adaptor proteins, such as myeloid differentiation primary response protein 88 (MyD88) and TIR-containing adapter-inducing interferon-β (TRIF), which subsequently activate downstream signals, including NF-κB, MAPKs, or interferon-regulatory factor (IRF). Activation of those signaling pathways promotes the transcription of pro-inflammatory cytokine genes, leading to the release of cytokines [91].

The activation of TLRs has been observed in DM. A significant increase in the expression of TLRs was observed in non-obese diabetic mice. Simultaneously, NF-κB and pro-inflammatory cytokines IL-6, IL-12, and TNF-α were significantly increased with downregulation of anti-inflammatory cytokine IL-10 [92]. TLR2-knockout also reduced the incidence of T1DM following repeated low-dose STZ injection in mice [93]. TLR2/6 and TLR4-activation in macrophages induced islet inflammation and disrupted the insulin gene expression in beta cells via increasing IL-1 and IL-6 [94].

Clinically, increased expression of TLR2 and 4 was also demonstrated in diabetic patients [95]. The *TLR4* gene expression in mononuclear cells of T2DM patients demonstrated a five-fold increase compared to non-diabetic controls [96]. In T1DM patients, gene expression of TLR2 and TLR4 on the monocyte cell surface was also upregulated [95].

TLR2 may regulate the polarization of inflammatory macrophages. In the peripheral nerves of T2DM mice, TLR2 antagonist CU-CPT22 repolarized M1 macrophages to M2 macrophages, decreased the release of cytokines, and ameliorated pain sensitivity in T2DM mice [97].

TLR4 may function as a prognostic biomarker for DSP. A significant increase in both mRNA and protein of TLR4 expressions in DSP was observed, and the increased levels of TNF-α and IL-6 correlated with TLR4 expression. The levels of TLR4 and TNF-α had a high accuracy in predicting the progression of neuropathy [98].

**Figure 5:**
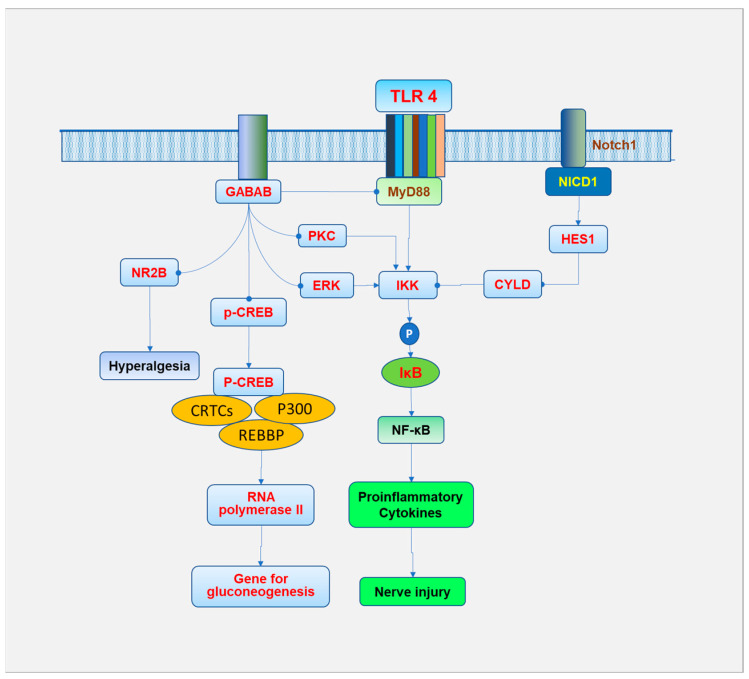
Role of TLR4 signaling pathway in DPN. During diabetes, increased expression of TLR4 recruits cytoplasmic TIR domain-containing adaptor proteins, such as myeloid differentiation primary response protein 88 (MyD88), followed by activation of IκB kinase (IKK), leading to activating nuclear factor (NF)-κB and subsequent release of proinflammatory cytokines. High glucose exposure induced the expression of Notch1 and hairy and enhancer of split 1 (HES1) and Notch1 intracellular domain (NICD1) in DRG neurons. HES1 is a downstream transcription factor of Notch1. Both TLR4 and Notch1 induce NF-κB through activating IKK, followed by phosphorylating IκB. HES1 activates IKK by inhibiting the cylindromatosis lysine 63 deubiquitinase (CYLD), a negative IKK complex regulator. Activation of TLR4 has been associated with the downregulation of the gamma-aminobutyric acid B (GABAB) receptor. Activation of GABAB receptors reduced the expression of phospho-cAMP-response element binding protein (p-CREB), N-methyl D-aspartate receptor subtype 2B (NR2B), extracellular signal-regulated kinase (ERK), and PKC. Both ERK and PKC can activate IKK. CREB in the nucleus binds to its cofactors, REB-binding protein (REBBP), P300, and CREB-regulated transcription co-activators (CRTCs), to form a complex, which interacts with RNA polymerase II to enhance the expression of the glucose production gene, promoting gluconeogenesis.

TLR4 has been shown to interact with Notch1 to regulate inflammatory response and neuropathic pain (Figure 5). High glucose exposure induced the expression of Notch1 mRNA, hairy and enhancer of split 1 (HES1) mRNA, TLR4 protein, and Notch1 intracellular domain (NICD1) in DRG neurons. The interaction between NICD1 and TLR4 in DRG neurons was upregulated after elevated glucose exposure. The mechanical allodynia and thermal hyperalgesia thresholds were significantly improved by either Notch1 or TLR4 inhibition with decreased levels of TNF-α in DRG from diabetic neuropathic rats [99]. Both TLR4 and Notch1 activate NF-κB through the IKK and I-κB pathway. HES1 activates IKK by inhibiting the cylindromatosis lysine 63 deubiquitinase, a negative IKK complex regulator [100]. TLR4-induced diabetic neuropathic pain is also relieved by glucose-6-phosphate dehydrogenase (G6PD). Significant decreases in G6PD and an increase in TLR4 were observed in the DRG of diabetic rats compared to age-matched control rats. Overexpression of G6PD by intrathecal injection markedly reduced the expression of TLR4 and attenuated pain hypersensitivity of diabetic rats [101].

The activation of TLR4 downregulates the gamma-aminobutyric acid B (GABAB) receptor. Inhibition of GABAB receptors significantly increased the expression of TLR4, Myd88, and NF-κB and reduced the levels of cytokines IL-1 and TNF-α in the spinal cord in DNP rats [102]. The anti-allodynic effects of GABAB agonist duloxetine in T1DM rats were observed to be accompanied by inhibition of the TLR4-Myd88 mediated pathway in the dorsal horn of the spinal cord [103]. Alternatively, baclofen activates the GABAB receptor and relieves diabetic neuropathic pain by decreasing the expression of phospho-cAMP-response element binding protein (p-CREB) and N-methyl D-aspartate receptor subtype 2B (NR2B) [104]. BHF177 increased the painful threshold by activating the GABAB receptors and subsequently suppressing PKC, CaMKII, p-ERK1/2, and p-CREB expressions in T1DM rats [105].

CREB, a transcription factor, is activated by phosphorylation, and p-CREB has been closely associated with developing chronic pain in neuropathy [106]. CREB has been involved in chemotherapy-induced neuropathic pain [107]. In addition, CREB in the nucleus binds to its cofactors, REB-binding protein (REBBP), P300, and CREB-regulated transcription co-activators (CRTCs), and forms a complex to regulate RNA polymerase II, resulting in upregulation of the glucose production gene and promoting gluconeogenesis [108]. CREB was reportedly involved in the transient receptor potential vanilloid 1 (TRPV1) induced pain pathway. TRPV induces calcium inward flow, leading to phosphorylating calcium/calmodulin-dependent protein kinase II (CaMKII) [109]. The overexpression of TRPV1 and CaMKII and activation of the downstream target CREB on the dorsal horn of the spinal cord in DNP rats was observed, which was reversed by capsazepine, a TRPV1 antagonist. Capsazepine treatment also downregulated the pain sensitivity of DSP in rats [110].

NR2B is a subunit of N-methyl-D-aspartate (NMDA) receptor and is involved in learning, memory, and pain perception [111]. Minocycline alleviates nociceptive response in rats via inhibiting NR2B in the spinal cord [112]. Ifenprodil treatment alleviates allodynia and nociceptive response by decreasing the expression of phosphorylated and total spinal NR2B in T1DM rats [113]. Intrathecal injection of Ro 25-6981 (selective NR2B antagonist) attenuated tactile allodynia in prediabetic T2DM mice [114].

The elevation of TLR9 in the spinal cords of STZ-injected rats and high glucose-treated rat microglia was also observed. TLR9 knockout attenuated the heat and mechanical hypersensitivity in T1DM rats. In addition, activation of TLR9 promotes the release of TNF-α, IL-1β, and IL-6 from microglia by activating p38MAPK pathway [115].

#### 3.2.5. Cytokines

Cytokines are small glycoproteins secreted mainly from immune cells and from non-neuronal glial cells in the nervous system. Immune cells are the primary source of IL-1, IL-6, IL-8, TNF-α, monocyte chemoattractant protein-1, and C-reactive protein (CRP). CRP is an annular pentameric protein and inflammatory marker, increasing plasma concentration during an inflammatory response. Cytokines function through binding to their receptors, activating intracellular signaling pathways, such as MAPK, NF-κB, Janus kinase (JAK)-signal transducer, and activator of transcription (STAT), further increasing the production of inflammatory mediators.

Chemokines are secreted cytokines crucial in recruiting leukocytes to the inflammatory sites. The subfamilies of chemokines are designated CC, CXC, CX3C, or XC based on the cysteine residues (assigned as C) on the sequence and other amino acids (assigned as X).

In addition, adhesion molecules, vasoactive amines and peptides (histamine, serotonin, bradykinin), arachidonic acid, and its derivatives (prostaglandins) also mediate the inflammatory process.

Cytokines play a critical essential role in developing diabetic neuropathy. In diabetic patients with a mean follow-up of 6.5 years aged 62–81 years of old Korean population, higher CRP, IL-6, TNF-α, IL-1 receptor antagonist (IL-1RA), and soluble intercellular adhesion molecule-1 (sICAM-1) and lower adiponectin levels were found in DSP in age- and sex-adjusted analysis [116]. A prospective study with over five years of observation indicated that elevated plasma levels of TNF-α and ICAM-1 increased the incident DSP after adjusting for known DSP risk factors in the Chinese population [117].

##### TNF-α

TNF-α is a crucial inflammatory marker for DSP. A meta-analysis including 14 studies involving 2,650 participants demonstrated that increased serum TNF-α levels in DSP patients were concluded [118]. A more complicated systemic and meta-review indicated 82.6% of studies showed an elevated serum TNF-α in DSP patients over none-DSP T2DM patients [119]. The increased levels of serum TNF-α were significantly related to an increased risk of DSP in T2DM patients. However, in some studies, TNF-α was reported to contribute to painful DSP [120]. TNF-α levels correlate with the severity and disability of DSP [121,122] and correlate with NCS [123]. In a recent case-control study, TNF-α has high predicting values for detecting DSP. The sensitivity and specificity of TNF-α were 95.7% and 61.4%, respectively [124].

##### IL-1

IL-1 is a critical cytokine in inflammatory response to various stimuli. IL-1 also induces a signaling cascade that increases the release of multiple cytokines and chemokines.

The involvement of IL-1 in the development of DSP has been extensively investigated. In T1DM patients with DSP, IL-1β receptor antagonist (IL-1RA) was shown to protect myelin and the axon of the sciatic nerve [125]. IL-1RA inhibits the pro-inflammatory action of IL-1β. Increased IL-1RA levels may be a defense response to IL-1β in DSP and other low-grade inflammatory diseases. Serum concentrations of IL-1RA positively correlate with DSP and higher MNSI scores [116]. In T2DM patients with DSP, a higher level of IL-1β in sciatic nerves than that in T2DM-only patients was also illustrated [126].

IL-1β has a role in neuropathic pain. IL-1β may induce the phosphorylation of NMDA receptors in neurons of the spinal dorsal horn and improve pain conduction in T2DM mice [127]. Clinical trials of recombinant IL-1RA and IL-1β inhibitors further suggest the role of IL-1 in the progression of DSP [116].

##### IL-6

IL-6 is a pleiotropic cytokine that can be an inflammatory biomarker for DSP. Increased plasma levels of IL-6 were demonstrated in Chinese diabetes patients with neuropathy [117]. In DSP patients, serum IL-6 is significantly associated with the MNSI score [116]. In T2DM patients, high serum IL-6 was associated with developing DSP and slowed motor NCV [128].

However, IL-6 may have anti-inflammatory properties in a condition-dependent manner. In experimental diabetes, IL-6 shows some beneficial effects. Subcutaneous IL-6 administration corrected thermal hyperalgesia and tactile allodynia, improved sciatic motor and saphenous sensory NCV, and increased sciatic endoneurial perfusion in T1DM rats [129,130]. However, reduced serum levels of IL-6 by liraglutide treatment in T1DM patients failed to improve the function of neurons at the central, autonomic, or peripheral levels [131]. In this regard, more studies should clarify the beneficial and harmful serum concentrations of IL-6 and the exact conditions that enable exogenous IL-6 to show therapeutic effects on DSP.

##### Chemokines

Chemokines play essential roles in the migration and recruitment of inflammatory cells. Locally released chemokines induce inflammatory responses by interacting with cell surface G-protein-coupled chemokine receptors.

The increased blood chemokines have been demonstrated in experimental DSP and are associated with neuropathic pain. In T1DM DSP, mRNA levels of CC motif ligand 2 (CCL2), CCL5, and CCL7 were significantly elevated in male mice. The increases in CCL8 and CCL12 were also observed in female mice. Administration of a dual chemokine receptor 2 (CCR2)/CCR5 antagonist, cenicriviroc, reduced the analgesic sensitivity in both male and female T1DM mice [132]. The pronociceptive effect of CCL2 in T1DM mice may be through CCR4 since the effect of CCL2 was diminished by injection of C021, a CCR4 antagonist [133]. The expression of the CCL1 protein and the CCR8 mRNA were also upregulated in the spinal dorsal of T1DM mice, and the downregulation of CCL1/CCR8 reduced neuropathic pain [134]. In a mixed T2DM model of mice with impaired beta-cell function, a more pronounced increase of CCL2-5 and 12 and CXCL2 and 9 in the culture medium of coccygeal functional spine units was observed [135]. CXCL2 expression significantly increased in rat sciatic nerves of T1DM-induced DSP and high glucose-induced Schwann cells. Functional CXCL2 diminishment prevents cell apoptosis and inflammation in vitro and in vivo by downregulating cleaved caspase 3/9, inactivating NLRP3 inflammasome, and reducing cytokines [136]. CXC chemokine receptor 4 (CXCR4) is expressed in a subset of DRG sensory neurons and is associated with painful diabetic neuropathy. In T2DM mice, numerous CXCR4-expressing inflammatory cells have been shown to infiltrate into the DRG. Intraperitoneal application of the AMD3100, a CXCR4 antagonist, attenuated painful neuropathy in diabetic animals [137].

Clinically, a meta-analysis showed an increase in multiple chemokines, including CCL1, 2, 4, 5, 11 and CXCL8, 10, 13 in T2DM patients when compared to controls [138]. Serum CCL11/Eotaxin-1 levels were also significantly upregulated in diabetic patients, and a further increase in DSP patients was observed. In addition, serum CCL11/Eotaxin-1 level was associated with NCV of the ulnar and sural nerve and the action potential of the sensory nerve [139]. Therapeutic trials should explore the effectiveness of chemokine antagonists in DSP.

##### Interferon (IFN)-γ

IFN-γ is a cytokine that may have pro-inflammatory properties in immunocytes and the target tissues. IFN-γ also activates macrophages to release inflammatory cytokines [140]. Knockdown of IFN-γ in NOD mice showed protection against T cell infiltration in the peripheral nerves [141]. Interestingly, IFN-γ inhibits the proliferation of Schwann cells by suppressing the mammalian target of rapamycin complex 1 (mTORC1) and subsequently downregulating the expression of Ras-related protein Rab-11 [142].

##### C-Reactive Protein (CRP)

CRP is the inflammatory biomarker produced in the liver, upregulated in response to pro-inflammatory cytokines. Smooth muscle cells, macrophages, lymphocytes, endothelial cells, and adipocytes can also produce CRP. The native CRP is primarily synthesized as a homopentameric protein, which dissociates into five monomers at sites of inflammation. The CRP monomers may act as a pro-inflammatory mediator that binds to the Fc gamma receptors (FcγRs), membrane-bound proteins, leading to the release of proinflammatory cytokines, such as TNF-α and IL-6, while native CRP may have anti-inflammatory effects [143].

CRP has been recognized as a stable predictor for developing T2DM [144]. The predicting value of CRP-to-albumin ratio has been investigated in T2DM patients. The ratios positively correlated with the body mass index (BMI), fasting glucose, serum creatinine, triglyceride, and LDL-cholesterol levels. Moreover, the CRP-to-albumin ratio was shown as an independent risk factor for DSP, and the accuracy of the ratios for predicting DSP is high, with a sensitivity of 78% and specificity of 73%, respectively [145]. In patients with amputation for a diabetic foot ulcer, an elevated serum CRP level can predict mortality since the preoperative/postoperative CRP differences were associated with increased mortality [146].

##### Adhesion Molecules

Common cell adhesion molecules include vascular cell adhesion molecule-1 (VCAM-1), ICAM-1, and selectins (E-selectin, L-selectin, and P-selectin). Activating cell adhesion molecules induces the production of pro-inflammatory cytokines and promotes leukocyte recruitment into diabetic tissues, playing a role in the progression of DSP. The levels of cell adhesion molecules and selectins were significantly increased in DSP patients. The regression analysis shows that VCAM-1 is linked to diabetic neuropathy [147]. A prospective study showed that diabetic patients who developed DSP had a higher plasma ICAM-1. Increased ICAM-1 level contributes to increased incidence of DSP in these diabetic patients, suggesting ICAM-1 may have predicting values for DSP development [117].

#### 3.2.6. Secretary Phospholipase A2 (sPLA2)

Regarding inflammation, sPLA2 is an interesting marker of neuroinflammation in diabetic neuropathy. PLA2 is responsible for hydrolyzing phosphatidylcholine into lysophosphatidylcholine and arachidonic acid, which can be catalyzed to produce prostaglandins and leukotrienes. sPLA2 activity has been demonstrated to increase in T2DM patients. This increased activity was associated with low-grade inflammation and endothelial activation [148]. Moreover, high glucose promotes the expression of sPLA2 activity and proliferative responses in Schwann cells [149,150]. The activity of sPLA2 was significantly increased in T1DM patients with cardiovascular disease, accompanied by low-grade inflammation and endothelial activation [149,150]. The downstream component of sPLA2, cyclooxygenase activity, has also been implicated in developing experimental DSP [151].

Our study demonstrated that the sPLA2 activity in urine was significantly increased in diabetic patients. Further increase in the activity of urine sPLA2 was observed in patients with DSP. Moreover, more patients in the DSP group than diabetic patients without DSP demonstrated elevated sPLA2 activity with NCS in more than two motor nerves into the demyelination range by the American Academy of Neurology (AAN) criteria [152]. Our study suggests that urine sPLA2 activity could be used to identify a subgroup of DSP with acquired demyelination in diabetic DSP.

The 8-iso-prostaglandin F2α (8-iso-PGF2α) is a prostaglandin that is a stable byproduct of lipid peroxides of arachidonic acid. The plasma 8-iso-PGF2α was significantly elevated in the DSP patients [153]. Urinary 8-iso-PGF2α excretion was also increased in T2DM patients. Another product of arachidonic acid thromboxane can further break down to produce 11-dehydro-thromboxane B2 (DTXB2). Urinary 8-iso-PGF2 levels linearly correlated with blood glucose and urinary DTXB2 in T2DM patients [154].

#### 3.2.7. Adiponectin

Adiponectin is a hormone released from adipose tissue, muscle, and the brain that increases insulin sensitivity and has anti-inflammatory properties. The levels of adiponectin in DSP and the link between them are inconsistent among studies. Either higher or lower adiponectin levels in patients with DSP have been illustrated. One clinical study indicated that increased high-molecular-weight (HMW) adiponectin and total adiponectin were associated with decreased motor NCV in T1DM and T2DM patients [128]. Both HMW and total adiponectin were associated with sensory NCV in T2DM patients. In a Chinese study, DSP patients had higher serum adiponectin levels, and serum adiponectin was positively associated with DSP in T2DM patients [155]. Some investigations did not show a significant association between adiponectin and DSP [156].

#### 3.2.8. Caveolin 1

Caveolin-1 is a membrane protein that has an anti-inflammatory property. In a clinical study of T2DM patients, increased expression of TLR4, MyD88, phosphorylated I-κB, TNF-α, and IL-6, but decreased levels of caveolin-1 and total I-κB in monocytes were observed. More importantly, plasma TLR4, TNF-α, and IL-6 were negatively correlated with caveolin-1 in patients with DSP, suggesting that decreased levels of caveolin-1 contribute to an increased DSP inflammatory response [157]. Knockout of caveolin-1 in T1DM mice significantly decreased the NCV of the motor nerve and the pain sensitivity. The diminished caveolin-1 impaired the NCV and led to mechanical hypoalgesia by promoting the phosphorylation of Erb-B2 receptor tyrosine kinase 2 (ERBB2) [158]. In T2DM rats, caveolin-1 can regulate diabetic neuropathic pain by directly interacting with TLR4 to phosphorylate NR2B in the spinal cord [159].

## 4. Neurological Biomarkers

### 4.1. Neurofilament Light Chains

Neurofilaments are protein polymers that include neurofilaments of heavy, medium, and light chains. Neurofilaments are part of the neuronal cytoskeleton and are present in the cytoplasm of neurons. Neurofilaments support the growth of axons and dendrites and are involved in nerve conduction [160]. Neurofilament light chain (NFL) protein predominantly expresses in large, myelinated axons. Upon axonal and neuronal injury, NFL is released to cerebrospinal fluid and plasma, which can be used to evaluate axonal loss. Increased NFL has been widely used to assess axonal damage in neurodegeneration and neuroaxonal injury in neurodegenerative disorders, such as Alzheimer’s disease, in both patients and animals [161,162].

Higher serum NFL levels were associated with DSP and correlated with motor and sensory NCS [163]. However, NFL levels were not consistent with current neuropathic pain severity [164].

NFL levels have been correlated with microstructural nerve integrity. Fractional anisotropy, a sensitive marker for microstructural nerve integrity, was impaired in T2DM patients with DSP, which correlated with the NCV of the tibial and peroneal motor and sural sensory nerves. Increased NFL levels in these patients correlated with decreased sciatic fractional anisotropy [165].

### 4.2. Meteorin-like

Meteorin-like is homologous to the neurotrophic factor meteorin, which plays crucial roles in the development and regeneration of neurons [166]. There is a negative association between serum meteorite-like and BMI, HbA1c, and renal functional parameters in T2DM patients [167]. Significantly lower levels of serum meteorite-like were observed in the DSP group, and serum meteorite-like levels were correlated with DSP [166].

### 4.3. Neuron Associated Proteins

In patients with diabetic neuropathy, a panel of 11 neuron-correlated proteins (TNFRSF12A, SCARB2, N2DL-2, SKR3, EFNA4, LAYN, CLM-1, CD38, UNC5, GFR-alpha-1, and JAM-B) has been positively related to the Toronto Clinical Scoring System [168]. These proteins, involved in processes such as inflammation, neuronal survival, axonal guidance, and membrane function, show promise as biomarkers for DSP. Their correlation with neuropathy severity suggests potential utility in diagnosis, disease progression monitoring, and differentiation between painful and painless forms of the condition. However, further validation through large-scale, multicenter studies is essential to confirm their sensitivity, specificity, and clinical relevance, paving the way for their integration into diagnostic workflows and personalized treatment strategies.

Among these proteins, TNFRSF12A (TNF receptor superfamily member 12A) regulates several processes, including axon extension, synaptic function, angiogenesis, extrinsic apoptotic signaling pathway, and cell migration [169,170].

Axon guidance proteins function to regulate embryonic axon guidance, neuron cell body migration, and synaptic plasticity. As an axon guidance protein, EFNA4 (ephrin-A4) directs axon growth during development and controls the plasticity of synaptic connections in adults [171]. UNC5 (uncoordinated-5), an axon guidance protein receptor, is expressed on the growth cone and binds to netrin. Netrin binds to UNC5 to regulate axon guidance, cell migration, neurogenesis, and morphogenesis of neural structures [172]. Repulsive guidance molecule-A (RGMA) is a glycosylphosphatidylinositol-anchored membrane protein that plays a role in guiding axons. RGMA is involved in the degeneration of cochlear synapses, inhibiting the synaptogenesis of auditory nerve fibers [173]. RGMA was also found to be positively associated with DSP [174].

Junctional adhesion molecules (JAMs) express at tight junctions of epithelial cells and endothelial cells and function to interact with postsynaptic density domain-containing scaffolding proteins and induce cell signaling pathways [175]. CD38 has been associated with immunomodulatory activities and is involved in the pathogenesis of osteoarthritis and associated pain [176]. As a GDNF family receptor alpha-1, GFR-alpha-1 expressed in the glial and peripheral immune system has been linked to neuroinflammation [177]. The exact mechanisms underlying their involvement in DSP should be further investigated.

In a group of the Korean population, CDH3 (cadherin-3), JAM-B, LAYN (layilin), and SCARA5 (scavenger receptor class a member 5) were also found to be positively associated with DSP in patients with diabetes [174]. CDH3 is a calcium-dependent cell adhesion protein that regulates tumor growth and cell migration and is associated with poor patient prognosis in glioblastoma [178]. CDH3 is also expressed in the developing nervous system. It may play a role in establishing the central nervous system’s cytoarchitecture and promoting axon elongation [179]. LAYN is a type 1 transmembrane protein that functions as a hyaluronic acid receptor and interacts with cytoskeletal proteins [180]. LAYN was also found in mitochondria and regulated mitochondrial dynamics, promoting mitochondrial fission by activating cyclin-dependent kinase 1 and dynamin-related protein 1 [181]. SCARA5 is one member of the scavenger receptor family that plays an important role in the innate immune activities of epithelial cells [182]. SCARA5 has been implicated in diabetic neuropathy, neuroinflammatory conditions, and cancers as a tumor suppressor [174,183,184].

In addition, serum levels of growth differentiation factor-15 (GDF-15) in T2DM patients have been identified as a possible biomarker for DSP. GDF15 is also called macrophage-inhibiting cytokine 1, belonging to a superfamily of transforming growth factor-beta. GDF-15 possesses anti-inflammatory activities and has been associated with various diseases, such as aging, metabolic diseases, heart failure, cancers, renal failure, diabetes mellitus, and mitochondrial disease [185,186].

The higher levels of GDF15 have been associated with diabetic complications [187]. As serum GDF-15 increases in T2DM patients, the DSP prevalence and BMI gradually increase, and motor and sensory NCV decrease. Linear regression analysis indicated that the GDF-15 levels were significantly associated with all latency and amplitude of sensory and motor nerves [188,189]. The accuracy of GDF-15 in predicting sensory and motor nerve neuropathy was compatible with HbA1c. In T1DM patients, a positive correlation between plasma GDF-15 and neuropathy impairment scores and high-sensitive CRP was also demonstrated [190].

### 4.4. Myelin Proteins

Myelin-producing Schwann cells maintain axonal myelination in the peripheral system. High glucose-induced cell signaling pathways lead to programmed cell death in Schwann cells [191]. During diabetes, high expression of aldose reductase and overactive polyol pathway in Schwann cells were observed [192].

Activation of mixed lineage kinase domain-like protein (MLKL), a core regulator of necroptosis, has been associated with myelin sheath breakdown in diabetic mice. Schwann cell S441A, single-amino acid knock-in mutation, or pharmacological inhibition, protected myelin sheath and improved NCV in STZ-induced diabetic mice [193]. In human biopsy samples from diabetic patients, the damage of the myelin sheaths of sural nerves was illustrated. Moreover, the activation of MLKL and the MLKL-induced myelin breakdown were observed in patients with diabetic neuropathy [193].

Myelin protein zero (MPZ) is an essential component of the myelin sheath. The levels of MPZ circulating mRNA significantly decreased in DSP patients. The downregulation of MPZ has 24 months in advanced predicting values for hypoalgesia and progressive nerve function loss [194].

Peripheral myelin protein 22 (PMP22) is also a crucial component of myelin sheath and is carbonylated and aggregated in sciatic nerves of db/db mice, which is oxidative stress associated. The results suggest that oxidation-induced PMP22 misfolding may mediate the demyelination and NCS in DSP [195].

## 5. Microvascular Biomarkers

The impairment of microvascular circulation, which affects the nerves in DSP, is one of the major players in the development of DSP.

### 5.1. Fibrinogen

Increased plasma fibrinogen levels in patients with T2DM significantly correlated with the occurrence of microvascular complications. In DSP patients, lower extremity perfusion was significantly impaired. The development of diabetic neuropathy was significantly correlated with increased fibrinogen levels [196].

### 5.2. C-Peptide

C-peptide is produced in the pancreas when insulin is secreted in equal amounts. C-peptide levels showed a positive correlation with impairment of lower extremity perfusion and a negative with HbA1c. A U-shape pattern of peripheral microcirculation and C-peptide levels was observed, showing surprisingly better circulation in the severe-DSP group than in the mild one [197].

### 5.3. VEGF

VEGF can increase vascular permeability and promote endothelial and mesangial cell growth and proliferation, impairing microcirculation and increasing extracellular matrix accumulation [198]. Significantly elevated VEGF levels in T2DM patients with neuropathy were observed compared to those without DSP. VEGF negatively correlated with motor nerve amplitude and positively correlated with the neuropathy symptom and diabetes neuropathy examination scores [156]. A systemic review demonstrated an increased VEGF level in DSP patients. However, no correlation was found between increased VEGF levels and an increased DSP risk [199].

### 5.4. NO

NO may play complicated roles in diabetes and DSP. NO can function as free radicals by forming peroxynitrite radicals to damage proteins and other molecules. In diabetic patients, plasma superoxide anion and peroxynitrite radicals were significantly increased, with further elevation in DSP patients. In multivariate models, plasma superoxide anion and peroxynitrite were independently associated with neuropathic deficits [153].

NO is also an endogenous vasodilator that benefits local blood flow to nerves. NO is synthesized by the reaction under NO synthases (NOSs). Three NOS isoforms have been identified, including neuronal NOS (nNOS), iNOS, and endothelial NOS (eNOS) [200]. The nNOS produces NO in the nervous system and plays a role in synaptic plasticity and microcirculation. The iNOS is induced by injury or pro-inflammatory response. High amounts of NO production by iNOS may exert harmful effects on the body and be implicated in several diseases, including diabetes and DSP. The eNOS may be critical in maintaining vascular dilation for local perfusion.

The downregulation of eNOS expression and reduced levels of the vasodilator NO in DSP patients have been observed. The deficiency of NO leads to reduced blood flow to the peripheral nerve, which leads to nerve injury [201]. Impaired NO synthesis has been implicated in diabetic painful neuropathy, and cutaneous application of NO-releasing reagents in subjects with DSP significantly reduced neuropathic pain [202]. In experimental diabetes, the production of AGEs can quench NO activity both in vitro and in vivo, impairing endothelium-dependent relaxation. Administration of aminoguanidine to inhibit AGE induction prevents NO quenching and ameliorates the vasodilation [203].

In experimental animals, the increase of eNOS in the lumbar DRG of rats with T1DM was observed [204]. High glucose treatment significantly and dose-dependently increased NO levels and eNOS and iNOS gene expression in HUVEC cells [205]. Serum NO level was also significantly higher in T2DM patients compared with control.

A systemic review of trials in both T1DM and T2DM patients of the European population demonstrated a significant increase in the levels of NOx (nitrate and nitrite, the stable NO products) [206]. However, significantly lower NO levels were found in diabetic subjects for more than five years [205], possibly reflecting NO consumption after a more extended period. The levels and roles of NO in developing DSP may depend on the disease’s severity and progression, and the value of NO to predict DSP has not yet been clarified.

## 6. MicroRNAs (miRNAs)

MicroRNAs (miRNAs) are non-coding small RNAs and play critical roles in developing and progressing diabetes-induced complications. Accumulated evidence indicates that the aberrant expression of miRNA can be involved in the development of diabetic neuropathy and neuropathic pain.

The miRNA-146a is one of the most investigated miRNAs. In rat models of both T1DM and T2DM, the expression of miRNA-146a in the sciatic nerve in the DSP group was significantly decreased [126,207]. The interesting finding is that the levels of miR-146a were negatively correlated with the concentrations of IL-1β, TNF-α, and NF-κB, suggesting that decreased levels of miR-146a were associated with increased inflammatory response [126]. Consisting with this indication, miRNA-146a mimics administration was reported to improve sensory and motor NCV of the sciatic nerve, increase regional blood flow in sciatic nerve tissues, and repress the activation of macrophages and pro-inflammatory gene expressions [208]. Further study indicated that miRNA-146a ameliorated DSP through thymosin-β4 (Tβ4) to promote axonal outgrowth and capillary-like tube formation [209]. Sildenafil, a phosphodiesterase type 5 inhibitor, improved DSP by enhancing miRNA-146a expression and decreasing the levels of IL-1 receptor-activated kinase (IRAK1) and TNF receptor-associated factor (TRAF) 6 levels in DRG neurons since blockage of miR-146a diminished the effects sildenafil on the expression of IRAK1 and TRAF6 and lost the protection of DRG neurons during hyperglycemia [210]. The predicting sensitivity and specificity of miRNA-146a for DSP in patients with T2DM were 83.7% and 60.8%, respectively [211]. Nano-miR-146a-5p has been demonstrated to improve NCV, attenuate the morphological damage, and increase the expression of myelin basic protein with reduced demyelination of the sciatic nerve in DSP of T2DM rats [212].

However, in T1DM rats, a threefold increase in the expression of miR-146a was observed in the sciatic nerve with an increase in the NF-κB activity and the levels of TNF-α, IL-6, and IL-1β in the sciatic nerve [213]. The difference is whether due to the transient acute response or dependent on the different pathogenesis, which requires further investigation.

The involvement of miRNA-34a-5p in DSP has been reported. In human microglia cells, knockdown of miRNA-34a-5p antagonized high glucose-induced microglia activation and inflammatory response by high glucose through targeting ectonucleotide pyrophosphatase/phosphodiesterase family member 3 (ENPP3), an extracellular enzyme that plays a key role in regulating inflammation and allergic responses [214].

However, in patients with DSP, serum miRNA-34a-5p was upregulated and positively correlated with the levels of triglyceride and HbAc1. The study identified miRNA-34a-5p as a risk factor for DNP and may serve as a biomarker for the early detection of DSP. The upregulation of miRNA-34a-5p was also a risk factor for diabetic neuropathic pain. In patients with painful diabetic neuropathy, the levels of miRNA-34a-5p were significantly higher compared with non-painful diabetic patients [214].

Another miRNA, miRNA-155 has been reported to regulate inflammatory response. The downregulation of miRNA-155 in sciatic nerves was observed in the db/db mice. In contrast, miR-155 mimics increased levels of miR-155 in both sciatic nerves and plasma, accompanied by increased blood flow in sciatic nerves and improvement of NCV, with suppression of multiple pro-inflammatory genes, including TRAF2, Notch2, and Sortilin (SORT1), and decreased levels of TNF-α, iNOS, IL-1β, and chitinase-like protein 3 (Ym1) [215]. COX-2 inhibitor celecoxib increased the expression of miR-155. Celecoxib protected DRG neuros from apoptosis under high glucose exposure, which were antagonized by a miR-155 inhibitor, suggesting that miR-155 interacts with COX-2 following celecoxib treatment [216].

In T2DM patients with DSP, blood levels of miRNA-155 in DSP patients were significantly decreased compared to the T2DM patients without DSP, while higher levels of miRNA-155 might serve as protective factors against DSP development. Multivariate logistic regression analysis indicated that the predicting sensitivity and specificity of miRNA-155 for DSP were 91.8% and 37.3%, respectively [211].

The miRNA-125a-5p in astrocytes reduces DSP pain in db/db mice. A decrease in miRNA-125a-5p was observed in sciatic nerve tissues in db/db mice and high glucose-treated astrocytes. Systemic administration of miRNA-125a-5p mimics increased mechanical allodynia and thermal hyperalgesia by decreasing the activation of astrocytes [217]. Similarly, miRNA-503-5p attenuated neuropathic pain in T2DM mice by downregulating septin-9 (SEPT9) to inhibit astrocyte activation [218].

MiRNA-184-5p represses neuropathic pain. In T1DM mice, miRNA-184-5p expression was significantly decreased in the spinal dorsal, while intrathecal administration of miRNA-184-5p agonist attenuated neuropathic pain in T1DM mice; in contrast, intrathecal miRNA-184-5p antagonist induces pain behaviors in naïve mice [134].

## 7. Genetic Variants and DSP

### 7.1. Keep1 Variants

Keap1 variants have been demonstrated to regulate susceptibility to diabetic neuropathy. Some Keap1 mutations have been found and may increase the interaction between Nrf2 and ARE [219]. For example, the Keap1 rs11085735 (A/C) polymorphism in intron 2 affects the Kelch-1 domain structure and impairs Keap1 and Nrf2 interaction. The Keap1 rs1048290 minor allele has been linked to the impairment in oxidative stress response since it caused higher expression of Keap1 and increased its association with Nrf2 [220].

The Keap1 (rs11085735) variant with a minor A allele of Keap1 is associated with lower Keap1 expression. In DSP patients, Keap1 AA genotype, not CC genotype, correlated with lower GPX activity. A higher expression of Keap1 AA genotype was found in DSP patients [221].

### 7.2. TLR4 Polymorphism

The TLR4 D299G polymorphism has been linked to increased risk for vascular complications and diabetic retinopathy in T2DM patients but not associated with increased incidence of diabetic neuropathy and nephropathy [222]. However, a report 12 years earlier showed that the TLR4 gene with D299G and T399I genotype gene was associated with a lower incidence of DSP in T2DM, but not in T1DM patients [223]. The difference may be due to the subject size, geographics, and study design. The polymorphism of TLR4 only occurs in about 10% of diabetic patients, which may also be a concern. Further study should be performed to elucidate the exact influence of polymorphism of TLR4 on the development of DSP.

### 7.3. IL-1β Variants

The genetic susceptibility of IL-1β rs16944/IL-1RA VNTR variants to DSP has been investigated in a Turkish cohort [224]. For IL-1β, IL-1β rs16944 CT genotype was higher in healthy control, IL-1β rs16944 CC was higher, and rs16944 TT was lower in DSP patients compared to controls. In T2DM patients, the IL-1β rs16944 CC genotype was a risk factor for developing DSP with an over threefold increase in frequency. For IL-1RA, IL-1RA VNTR a1/a1 and a2/a2 genotypes were lower, and a1/a2 genotype was higher in DSP patients. The patients carrying the IL-1RA VNTR a1/T haplotype had a decreased risk of DSP than the control groups. The a1/a2 genotype was beneficial with lower total cholesterol in carriers; in contrast, the a2/a2 genotype contributed to a lower HDL level and higher total cholesterol level in patients.

### 7.4. NAD^+^ Metabolism-Related Genes

Gene analysis indicates that NAD^+^ metabolism-related genes (NMRGs) may play a role in DSP. ENPP3 is a glycoprotein expressed in various tissues and plays a role in limiting the response of mast cells and basophils during inflammation and in the metabolism of extracellular nucleotides. Nudix hydrolase 1 (NUDT12) promotes the decay of NAD-capped RNAs in cells [225]. ENPP3 and NUDT12 were upregulated in DSP samples [226]. The enrichment analysis demonstrated that ENPP3 was enriched in the TLRs pathway, and NUDT12 was enriched in young T2DM diabetes and insulin pathways [226]. However, the predicting values of ENPP3 and NUDT12 have not been illustrated, and further investigation is required to determine their prognostic values.

### 7.5. Others

Methylenetetrahydrofolate reductase (MTHFR) (rs1801133) and GPX (rs1050450) polymorphisms have been the risk factor of T2DM, diabetic neuropathy, and diabetic retinopathy. MTHFR TT genotype is negatively correlated with the levels of total oxidative stress and is associated with a 3.9-fold increased risk of neuropathy [227]. Patients with the GPX TT genotype had higher MDA levels and lower total oxidative stress levels. The frequencies of the T allele and TT genotype of GPX were significantly higher in DSP patients than those without DSP. The polymorphism in the GPX (rs1050450, 198CT) gene is associated with reduced antioxidant activity [228].

IFNγ 874 polymorphisms have been found in the South Indian population. The IFNγ 874AA genotype in T2DM patients with DSP occurs higher than controls. No significant rates were found for genotypes A/T and T/T genotype [229].

Among the IL-10 1082 G/A polymorphism, G/A, G/G, and A/A, only G/G genotype in the DNP patients group showed significant association with DSP compared to controls [229].

Two adiponectin polymorphisms, rs2241766+45T/G and rs1501299+276G/T, have been investigated in DSP. The different frequencies of TT, TG, and GG genotypes and the T and G alleles of T45G and G276T polymorphisms between DSP and none-DSP groups were observed. T45G and G276T polymorphisms result in reduced plasma levels of adiponectin in DSP and T2DM-only groups. The results suggest that the T45G and G276T polymorphisms correlate with an increased DSP risk in T2DM patients. TG haplotype also positively correlated with risk of DSP; in contrast, GG and GT haplotypes showed a negative relationship with DSP in T2DM patients [230].

## 8. Conclusions and Prospectives

The pathogenesis of DSP is complicated, and multiple pathological mechanisms are involved in its development, such as aberrant glucose metabolisms and associated oxidative stress and inflammation induction. The pathological processes produce changes in the proteins and molecules in the body, which may emerge in blood and other compartments to be determined as biomarkers (Table 1).

The current biomarkers for DSP have limitations. A marker’s values for predicting DSP depend on its accuracy, feasible features, and accessibility for determination. However, the biomarkers’ values in predicting DSP development are far from accurate. The sensitivity and specificity have a wide variety. In addition, blood biomarkers are challenging to differentiate the central from peripheral nerve degeneration. Moreover, different biomarkers may reflect the pathogenic progress in different phases.

However, the perspective of the application of biomarkers is bright. As discussed above, a single marker’s value in the DSP prognosis is limited. The combination of multiple biomarkers may be promising for accurately predicting DSP. In this regard, establishing a machine-learning platform that integrates different functional biomarkers may pave the way to success. Rapid progress in artificial intelligence (AI) will become an invaluable tool in clinical diagnosis. With the help of AI, the integration of various biomarkers to predict the progression of DSP will be anticipated shortly. The DSP predicting platform will most likely be established to serve clinicians in the next few years.

## Figures and Tables

**Table 1:** Roles of biomarkers for DSP.

Biomarkers	Roles	References
**Oxidative Stress**		
Thiol/Disulfide	The thiol-disulfide balance was impaired in DSP patients, with a decrease in native thiol and total thiol levels and an increase in disulfide levels.	[42,43]
Malondialdehyde	Lipid peroxidation product and one of the most used biomarkers for oxidative stress. The serum levels significantly increased and were correlated with the nerve deficit score, calculated by the Michigan Neuropathy Screening Instrument.	[46]
Poly (ADP-ribose) polymerase (PARP)	PARP is critical in DNA repair, mitochondrial homeostasis, and apoptosis. Hyperglycemia induces oxidative stress, resulting in overproduction of ROSs and activation of PARP. The hyperactivated PARP depletes NAD+ and ATP and causes the accumulation of ADP-ribose polymer, disrupts glycolysis, and impairs mitochondrial respiration, leading to ATP depletion and cell death. PARP also triggers the activation of NF-κB and subsequent release of proinflammatory cytokines.	[57,61,231]
**Inflammation**		
Macrophage	Macrophage infiltration, pro-inflammatory polarization, and inflammatory cytokine release in nerves contribute to nerve injury during diabetes.	[73,74,78]
Neutrophil-to-lymphocyte ratio (NLR)	NLR is an independent risk factor for DSP. NLR levels were significantly increased in the diabetic patients and correlated with the development of DSP. The accuracy of NLR in predicting the onset of DSP is 65.41%. In addition, HbA1c and NLR have some synergic effects on predicting DSP.	[81,82]
NF-κB	NF-κB is an integral signal regulating the activating cascade of gene transcription of pro-inflammatory cytokines. Long-term hyperglycemia increases the production of AGEs, which interact with receptors for AGEs (RAGE) to induce intracellular signaling pathways, increasing proinflammatory cytokine release, Higher serum NF-κB levels positively correlated with total neuropathy score in the DPN patients.	[67,68,69,70,84]
Toll-like receptors (TLRs)	TLRs interact with MyD88 and TRIF to activate NF-κB, MAPKs, or IRF to regulate inflammatory response. The expression of TLRs in peripheral blood cells was significantly increased with the elevation of NF-κB and pro-inflammatory cytokines in experimental diabetes. TLR2/6 and TLR4-activated macrophages contribute to islet inflammation and impair beta-cell insulin gene expression. Both TLR4 mRNA and protein expressions increased significantly in DSP compared to diabetes without DSP, which correlates with the levels of TNF-α and IL-6. In particular, TLR4 elevation is a risk factor for DSP and could be used to predict the progression of DSP.	[91,92,94,98]
TNF-α	TNF-α is a critical inflammatory cytokine in diabetes and DSP, and the elevated level of serum TNF-α was significantly associated with an increased risk of DSP. Higher TNF-α levels correlate with nerve damage severity, NCS, and disability. The sensitivity and specificity of TNF-α for predicting DSP were up to 95.7% and 61.4%, respectively.	[119,121,122,123,124]
IL-1β	IL-1 is a critical cytokine mediating the inflammatory response and induces a signaling cascade that increases the production of multiple cytokines and chemokines. A higher level of IL-1β in the sciatic nerves and spinal cord is associated with inflammation and neuropathic pain.	[126,127]
IL-6	IL-6 is a pleiotropic cytokine that can be an inflammatory biomarker for DSP. Elevated serum IL-6 is significantly associated with the MNSI score in DSP patients’ motor conduction slowing. The beneficial effects of IL-6 are controversial and may depend on the IL-6 levels and DSP conditions.	[116,128,129,130,131]
Chemokines	Increased mRNA levels of CC motif ligand 1 (CCL1), CCL2, CCL5, CCL7, CCL8, and CCL12 have been associated with analgesic sensitivity in DSP. CXCL2 expression significantly increased in rat sciatic nerves of T1DM-induced DSP and high glucose-induced Schwann cells. CXCL2 is associated with induction of the expression of cleaved caspase 3/9, activation of NLRP3 inflammasome, and release of pro-inflammatory cytokines. CXC chemokine receptor 4 (CXCR4) in a subset of DRG sensory neurons has been associated with painful diabetic neuropathy. Increased serum CCL11/Eotaxin-1 levels were correlated with nerve conduction velocity of the peripheral nerves and the action potential of the sensory nerve.	[132,134,136,137,139]
Adhesion molecules	The levels of adhesion molecules are significantly increased, which induces the release of pro-inflammatory cytokines and promotes leukocyte recruitment and infiltration into diabetic tissues. Higher levels of VCAM-1 and ICAM-1 have been associated with the development of diabetic neuropathy.	[117,147]
Secretary phospholipase A2 (sPLA2)	sPLA2 catalyzes phospholipids to release arachidonic acid, which then is converted to produce prostaglandins and leukotrienes, which play a role in inflammatory responses. sPLA2 and its downstream component cyclooxygenase activity have also been implicated in the development of experimental DSP. Elevated urine sPLA2 activity was correlated with NCS in DSP patients. The 8-iso-PGF2α and TXB2 are the two stable byproducts of lipid peroxides of arachidonic acid, which are significantly increased in the urine of DSP patients and may be used as a predictor for DSP.	[151,152,154]
Adiponectin	Adiponectin functions to increase insulin sensitivity and prevent inflammatory response. The level changes and functions in DSP are not consistent. Some studies demonstrated that higher adiponectin levels are positively associated with DSP in T2DM patients.	[155,156]
Interferon (IFN)-γ	IFN-γ may have pro-inflammatory effects and activate macrophages to release inflammatory cytokines. IFN-γ has been shown to contribute to the development of neuropathy in nonobese diabetic mice, induce T cell infiltration in the peripheral nerves, and inhibit Schwann cells.	[140,141,142]
Caveolin 1	Caveolin-1 is a membrane protein that plays an anti-inflammatory role. In diabetes, lower plasma levels of caveolin-1 were negatively correlated with plasma concentrations of TLR4, TNF-α, and IL-6. Low caveolin-1 levels were also associated with NCS and mechanical hypoalgesia.	[157,158]
C-reactive protein (CRP)	CRP is the inflammatory response biomarker. The CRP-to-albumin ratio has been positively correlated with BMI, fasting glucose, serum creatinine, triglyceride, and LDL-cholesterol levels and was negatively associated with the estimated glomerular filtration rate. CRP-to-albumin ratio has been considered an independent risk factor for DSP, and the accuracy of the ratios for predicting DSP is high, with a sensitivity of 78% and specificity of 73%, respectively.	[144,145]
**Neurological Biomarkers**		
Neurofilament light chains (NFL)	Blood NFL can be used to evaluate axonal loss. Higher serum NFL levels were correlated with motor and sensory NCS and microstructural nerve integrity.	[163,165]
Meteorin-like	Meteorin-like is a secreted protein homologous to the neurotrophic factor meteorin and regulates the development, maintenance, and regeneration of neurons. Serum meteorite-like were significantly decreased in DSP patients, and its level was correlated with the severity of DSP.	[166]
Neuron associated proteins	These biomarkers involve inflammation, neuronal survival, axonal guidance, and membrane function. Their levels are positively associated with the severity of diabetic neuropathy.	[168]
	Axon guidance proteins, EFNA4, UNC5, and RGMA, regulate embryonic axon guidance, neuron cell body migration, and synaptic plasticity.	[171,172,174]
	TNFRSF12A regulates axon extension, synaptic function, angiogenesis, apoptotic signaling pathway, and cell migration.	[169,170]
	JAMs interact with postsynaptic density domain-containing scaffolding proteins and induce cell signaling pathways.	[175]
	GFR-alpha-1 expressed in glial and peripheral immune system has been linked to neuroinflammation.	[177]
	CDH3, LAYN, and SCARA5 are all positively associated with DSP in patients with diabetes. CDH3 is a calcium-dependent cell adhesion protein expressed in the developing nervous system. It may help to establish cytoarchitecture in the central nervous system and regulate axon elongation. LAYN was also found in mitochondria and regulated mitochondrial dynamics, promoting mitochondrial fission. SCARA5 is one member of the scavenger receptor family that plays an important role in innate immune activities.	[174,179,181,182]
Growth differentiation factor-15 (GDF-15)	GDF15, also called macrophage-inhibiting cytokine 1, belongs to a superfamily of transforming growth factor-beta and has anti-inflammatory activities. Increased levels of GDF-15 significantly correlated with all latency and amplitude of sensory and motor nerves, as well as F-wave and H-reflex latencies. In T1DM patients, plasma GDF-15 positive correlates with neuropathy impairment scores and high-sensitive CRP.	[187,188,189,190]
Myelin proteins	Myelin protein zero (MPZ) was decreased in DSP, and decreased levels of MPZ have predicted values for hypoalgesia and progressive nerve function loss. Peripheral myelin protein 22 (PMP22): Oxidation stress-induced PMP22 misfolding and aggregation contribute to demyelination in DSP.	[194,195]
**Microvascular Biomarkers**		
Fibrinogen	Elevated plasma fibrinogen levels in patients with T2DM significantly correlated with the progression of diabetic neuropathy.	[196]
C-peptide	C-peptide is produced in the pancreas simultaneously with insulin secretion. C-peptide levels positively correlated with impairment of lower extremity perfusion and a negative association with HbA1c values.	[197]
VEGF	VEGF can increase vascular permeability and impair microcirculation. VEGF levels were increased in T2DM patients with neuropathy, negatively correlated with motor nerve amplitude, and positively correlated with the neuropathy symptom score and diabetes neuropathy examination score.	[156,198]
Nitric oxide (NO)	NO can function as free radicals by forming peroxynitrite radicals to damage proteins and other molecules. Plasma superoxide anion and peroxynitrite were independently associated with neuropathic deficits. NO is also an endogenous vasodilator that benefits local blood flow to nerves, and the deficiency of NO leads to reduced blood flow to the peripheral nerve. Impaired NO synthesis has been implicated in diabetic painful neuropathy. However, either increased or decreased serum NO has been observed in diabetic patients.	[153,201,202,205,206]
**miRNA**		
miRNA-146a	Downregulation in sciatic nerves and blood is associated with increased TNF-α, IL-1β, and NF-κB in sciatic nerve tissues of the DSP rats with T2DM.	[126,211]
	Upregulation in sciatic nerves of DSP with T1DM in rats with increased NF-κB activity and the concentration of TNF-α, IL-6, and IL-1β in the sciatic nerves.	[213]
miRNA-34a-5p	Upregulation in the serum of DSP patients positively correlates with the levels of triglyceride, fasting blood glucose, and glycated hemoglobin of DNP patients.	[110]
miRNA-155	miRNA-155 improves the blood flow in sciatic nerves and NCV and suppresses multiple proinflammatory genes. miRNA-155 was downregulated in sciatic nerves of DSP in db/db mice and blood in T2DM patients with DSP.	[211,215]
miRNA-184-5p	Downregulation in the spinal cord in T1DM mice with DSP	[134]
**Genetic Variants**		
Keep1rs11085735 Keap1 rs1048290	Keap1 variants affect oxidative stress and the susceptibility to diabetic neuropathy. Keap1 rs11085735 (A/C) polymorphism affects the Kelch-1 domain structure and impairs Keap1 and Nrf2 interaction. The Keap1 rs1048290 minor allele has an impaired response to oxidative stress, causing higher expression of Keap1 and increased association with Nrf2.	[220]
TLR4D299G	The TLR4 D299G polymorphism has been linked to an increased risk for vascular complications and diabetic retinopathy in T2DM patients. TLR4 gene with D299G and T399I genotype was associated with a lower incidence of DSP in T2DM.	[222,223]
IL-1β rs16944	IL-1β rs16944 CC genotype was a risk factor for developing DSP In T2DM patients.	[224]
NAD^+^ metabolism-related genes	ENPP3 is a glycoprotein and controls the response of mast cells and basophils during inflammation. NUDT12 promotes the decay of NAD-capped RNAs in cells. ENPP3 and NUDT12 were upregulated in DSP samples.	[225,226]
MTHFR, rs1801133 TT	Increases the risk of neuropathy in diabetic patients	[227]
GPX, rs1050450, T	GPX rs1050450, TT genotype was significantly higher in DSP patients. The GPX rs1050450, 198CT gene polymorphism is associated with increased oxidative stress.	[228]
IFNγ874AA	IFNγ 874AA genotype occurs higher in T2DM patients with DSP,	[229]
IL-10 1082 GG	IL-10 1082 G/G genotype has been associated with DSP.	[229]
Adiponectin T45G and G276T	T45G and G276T polymorphisms correlate with an increased DSP risk in T2DM patients. TG haplotype also positively, but GG and GT haplotypes negatively correlate with DSP in T2DM patients.	[230]

CCR8, C-C motif ligand1/8; CDH3, cadherin-3; DSP, diabetic sensorimotor polyneuropathy; DTXB2, 11-dehydro-thromboxane B2; EFNA4, ephrin-A4; GDF-15, growth differentiation factor-15; 8-iso-PGF2α, 8 -iso-prostaglandin F2α; IRF, interferon-regulatory factor; JAMs, junctional adhesion molecules; MTHFR, methylenetetrahydrofolate reductase; MyD88, myeloid differentiation primary response protein 88; NCS, nerve conduction slowing; NF-kB, nuclear factor kappa B; RGMA, repulsive guidance molecule-A; Tβ4, thymosin-β4; TRAF6, tumor necrosis factor (TNFR)-associated factor 6; TRIF, TIR-containing adapter-inducing interferon-β; UNC5, uncoordinated-5.

## Data Availability

No new data were created or analyzed in this study.
